# Review of Radiation Embrittlement of Aluminum Alloys Used in Research Reactors

**DOI:** 10.3390/ma18225236

**Published:** 2025-11-19

**Authors:** Ferenc Gillemot, Murthy Kolluri, Ildiko Szenthe, Frideriki Naziris, Lajos Berzy

**Affiliations:** 1 HUN-REN Centre for Energy Research, 1121 Budapest, Hungary; szenthe.ildiko@ek.hun-ren.hu (I.S.); berzy.lajos@ek.hun-ren.hu (L.B.); 2 NRG PALLAS, 1755 Petten, The Netherlands; kolluri@nrg.eu (M.K.); naziris@nrg.eu (F.N.)

**Keywords:** aluminum research reactors, radiation degradation, operation lifetime, transmutation

## Abstract

Research reactors are generally built from aluminum alloys during the last century. Most of the operation times of them already exceed the design lifetime. The original mechanical properties change during service. The radiation embrittlement affects them through three mechanisms: the transmutation of aluminum to silicon caused by thermal neutrons, gases (hydrogen, helium) occur by fast neutron transmutation, causing swelling, and matrix defects (dislocations, voids) occur by fast neutron irradiation. This paper summarizes the existing knowledge of the radiation degradation of aluminum alloys.

## 1. Introduction

Most research reactors (RRs) in operation today were built 30–60 years ago. These reactors mainly serve three purposes: producing isotopes, supplying neutron beams for research, and educating physics and engineering students. Since these tasks do not require high temperature and pressure, research reactors are typically pool or tank type structures operating at atmospheric pressure and room temperature or slightly above.

Depending on the country specific regulations, the construction of the RRs’ aging monitoring and use of surveillance specimen sets were not always required. Following the methodologies used for power reactors, presently the safety authorities request proper aging management of the RRs in the context of long-term operation; however, only few of them have dedicated aging management programs including surveillance programs (specimens for aging monitoring). The aging management programs of RRs are reactor specific and generally try to follow the methodology of power reactors. Detailed information on the structure of a good aging management program is given in the IAEA SG10 document [1]. Another example is the surveillance program of HFR (High Flux Reactor, Joint Research Center Institute of Energy Petten, The Netherlands) summarized in the paper of Luzginova [2].

Many RRs have exceeded their designed operational period, so it is important to consider and re-evaluate the material degradation of safety-critical components (SCCs). These low-temperature water-cooled and moderated RRs typically employ AlMg alloys (ASTM 5xxx series) and AlMgSi alloys (ASTM 6xxx series) as materials for the vessel and in-core components due to their good mechanical properties, corrosion resistance in water, excellent thermal conductivity, and lower degradation rate during high fluence neutron irradiation compared to steels. In addition, aluminum produces relatively short-lived radioactive isotopes when irradiated, simplifying operation and maintenance. Another advantage is their low neutron absorption coefficient, which minimizes beam flux loss—important for building research beam channels.

Throughout the lifetime of RRs, these materials are exposed to large thermal neutron fluence values (in some cases even up to ~10^27^ n/m^2^) [2]. Substantial damage to the material’s microstructure and mechanical properties can occur at these high fluence conditions, primarily due to the transmutation of aluminum into silicon by thermal neutrons and displacement damage due to fast neutrons [3,4].

The irradiation behavior of various aluminum alloys up to thermal fluence values of ~10–40 10^26^ n/m^2^ have been previously published [5,6,7,8,9]. However, there is a limited understanding of the materials’ degradation mechanisms and insufficient material data (specifically fracture toughness data) are available for high fluence values. In the literature, indications of an abrupt transition towards brittle behavior at higher fluences were found [5] and the degradation rates and contributing irradiation damage mechanisms are known to depend on the chemical composition of the alloys (5xxx or 6xxx series) [10]. For the development of accurate radiation damage prediction models, a deeper fundamental understanding and more mechanical testing data (tensile properties, fracture toughness, etc.) and microstructural investigations of irradiated RR materials are necessary.

An ongoing European research project, MAGIC RR, is initiated to improve the current understanding of the irradiation-induced mechanisms in aluminum alloys, investigate the applicability of sub-size specimen testing for surveillance testing, and to collect experiences on aging management for RRs [11]. As a starting point of this effort, a study of the literature is performed and information is collected on the aging of RR materials during service conditions. This paper summarizes the collected knowledge.

## 2. Properties of Aluminum Alloys Used for Research Reactors

The 5xxx and 6xxx alloys are relatively ductile in nature and have face-centered cubic (fcc) structures. Up to 1.8% Mg can be dissolved in aluminum, forming a eutectic structure. Excess Mg increases material strength without significantly decreasing toughness or elongation. Silicon is practically insoluble in aluminum at room temperature or below. Table 1 shows the chemical composition of the main alloying elements in alloys used for SCCs in RRs.

### 2.1. Mechanical Properties of As Received Aluminum Alloys

The mechanical properties of aluminum alloys vary widely based on production technology. Some alloys can be temper hardened. For research reactor structures, plates, bars, and pipes are commonly used. Table 2 shows the typical mechanical properties of mild and tempered alloys [12]. There are slight differences in mechanical properties depending on the manufacturer.

The welding mechanical properties of aluminum alloys vary widely based on filler material and welding technology. Reference values can be found in the literature and filler material manufacturer catalogs [13]. Most research reactors have been built using TIG (Tungsten Inert Gas) welding [14].

Due to the high ductility and medium strength of the mild as received aluminum alloys, the plane strain fracture toughness (K_1c_) of these alloys often cannot be directly measured, especially on thin plates. K_q_ (quasi values) are measured on 60–120 mm thick materials, providing values between 30 and 50 MPa√m [15]. These values are conservative and can be used for safety and lifetime evaluations of thinner-walled structures. In the case of embrittled aluminum alloys, fracture toughness tests may give valid values.

### 2.2. Property Correlation Method to Approximate Fracture Toughness

**Figure 1:**
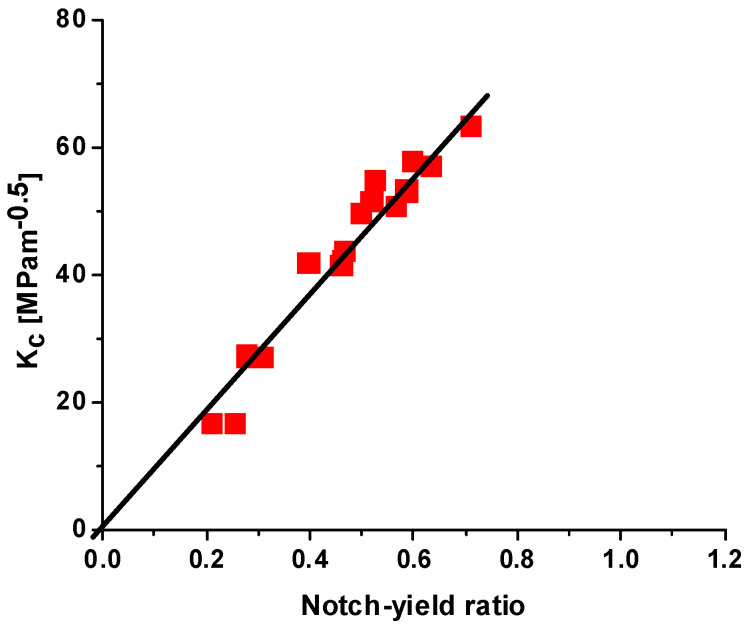
Correlation between the critical stress intensity factor and the notch–yield ratio for aluminum alloys [16,17].

The notch–yield ratio (the ratio of tensile strength of a notched specimen to the yield strength of an un-notched specimen) can determine the fracture toughness of aluminum alloys when direct K_1c_ measurements are not available (see Figure 1) [16,17,18,19].

## 3. Aged Properties

Lifetime evaluation of RRs must consider the aging of aluminum alloys to meet safety requirements. The main degradation mechanisms in RRs are radiation embrittlement, corrosion, and thermal aging (precipitation hardening) which occurs even at ambient temperatures.

### 3.1. Microstructural Effects of Radiation Aging

Radiation causes three typical aging mechanisms in aluminum alloys:

1. Transmutation of aluminum to silicon caused by thermal neutrons;
2. Gases (hydrogen, helium) occur by fast neutron transmutation, causing swelling;
3. Matrix defects (dislocations, voids) occur by fast neutron irradiation.

### 3.2. Al-Si Transmutation

Thermal neutrons cause the transmutation of Al into Si through the following sequential reactions:

^27^Al(n,γ) ^28^Al, ^28^Al → ^28^Si + β

This leads to an increase in Si content with increasing thermal neutron fluence. Transmutation-produced Si by thermal neutrons (generally neutrons E < 0.625 eV) causes substantial radiation damage in Al alloys. Kapusta et al. [9] confirmed that the Si content is a major indicator for the neutron irradiation effects. As the cross-section for neutron absorption on ^27^Al presents resonances in the epithermal energies range, the epithermal flux contributes significantly to silicon production.

A rough estimate of the irradiation-induced silicon content can be made by integrating the neutron fluence between 0 and 0.625 eV and normalizing it for E = 0.0254 eV. A thermal fluence of 1 × 10^26^ n/m^2^ (E = 0.0254 eV) produces 0.236 weight % silicon [10]. Irradiation of the 5154-0 (ALMg3) alloy in the High Flux Reactor (HFR) validated this correlation [11].

**Figure 2:**
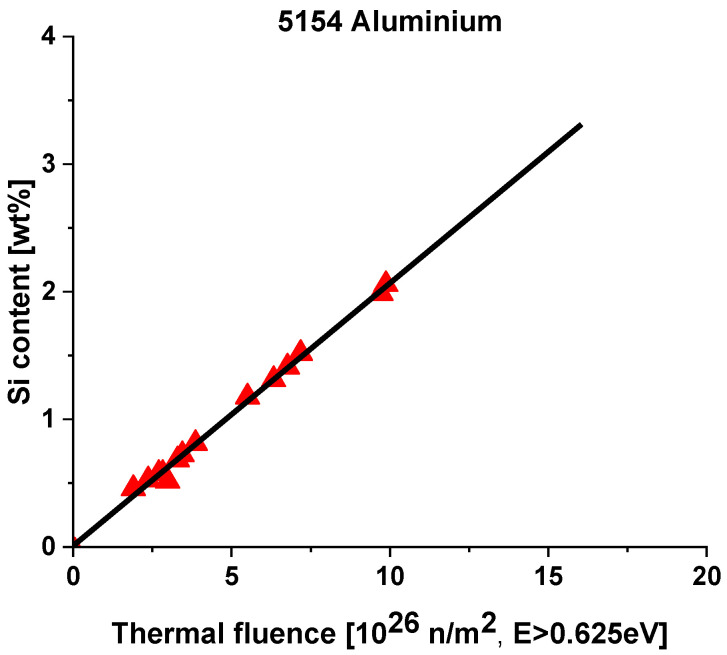
Silicon content increase in 5154-0 (AlMg3) alloy at room temperature. The diagram is valid for AlMg5, too [11].

Temperature significantly affects the thermal neutron spectra. If neutrons are cooled from room temperature to cryogenic, the normalized (E = 0.0254 eV) flux value becomes about 50% higher than at room temperature. In the discussion of the effect of radiation on the mechanical properties (Section 3.6), several figures show the embrittlement rate in the function of the thermal fluence. From the thermal fluence value, the silicon content can be calculated using Figure 2 [11].

### 3.3. Microstructural Consequences of Al-Si Transmutation

The aluminum lattice is face-centered cubic, with a lattice parameter of about 4 Angstroms. The magnesium cell is hexagonal, with edges being 3.2 and 5.2 Angstroms. Its solubility in aluminum is 1.8% at room temperature. When magnesium exceeds 1.8% in AlMg alloys, small, dispersed particles are precipitated throughout the matrix. Since the atomic radius of magnesium is slightly larger than that of aluminum, its incorporation into the aluminum lattice induces lattice strain, which can enhance the material’s strength properties.

The silicon lattice is cubic, with a cell edge of about 5.4 Angstroms. Silicon is practically non-soluble in aluminum at room temperature or below. As the silicon lattice parameter is larger than the aluminum lattice parameter, increasing silicon content increases the silicon precipitates and causes considerable hardening of AlSi alloys.

In AlMg alloys (series 5xxx), magnesium and irradiation-produced silicon become the Mg_2_Si phase, containing 63.2% magnesium and 36.8% silicon. The Mg_2_Si cell can be face-centered cubic, with edges being 6.35 Angstroms, or hexagonal, with edges being 7.05 and 4.05 Angstroms. It also stresses the aluminum lattice and contributes to the hardening.

The 6xxx series of aluminum alloys also contain Si and Mg. The Si content is less than 1%, and the Mg content is less than 1.5% in all 6xxx as received standard alloys. They are precipitation-hardening metals.

If silicon content increases due to radiation in AlMg alloys, the Mg_2_Si phase will consume nearly all Mg, causing embrittlement. If silicon content becomes higher than half of the Mg content, excess silicon is expected to precipitate around Mg_2_Si clusters, grain boundaries, and the Al matrix. These precipitates will further increase embrittlement.

Fast neutron fluence damages the matrix and causes dislocations. Dislocations act as nucleation sites for Mg_2_Si and Si precipitations. The size of precipitations depends on thermal fluence, on the number of precipitations, and on the thermal and fast flux ratio.

The series of 5xxx alloys first transform to series 6xxx (AlMgSi) alloys by transmutation. If the magnesium content is originally high (e.g., 4.5–6% in AlMg5), the quantity of the Mg_2_Si phase will be 2–3 times more than in the 6000 series. At about 2–2.5% silicon content, all Mg is consumed, and further irradiation will result in silicon precipitates. On the grain boundaries, the number of precipitations is higher than in the matrix. The Mg_2_Si and Si precipitates formed at high fluences may act as crack initiating sites during fracture. The fracture surface is characterized by dominant micro dimples and some cleavage facets [10]. The mixed-mode fracture surface (ductile dimples and brittle facets) proves that the material still has considerable toughness, but further irradiation may cause serious embrittlement.

**Figure 3:**
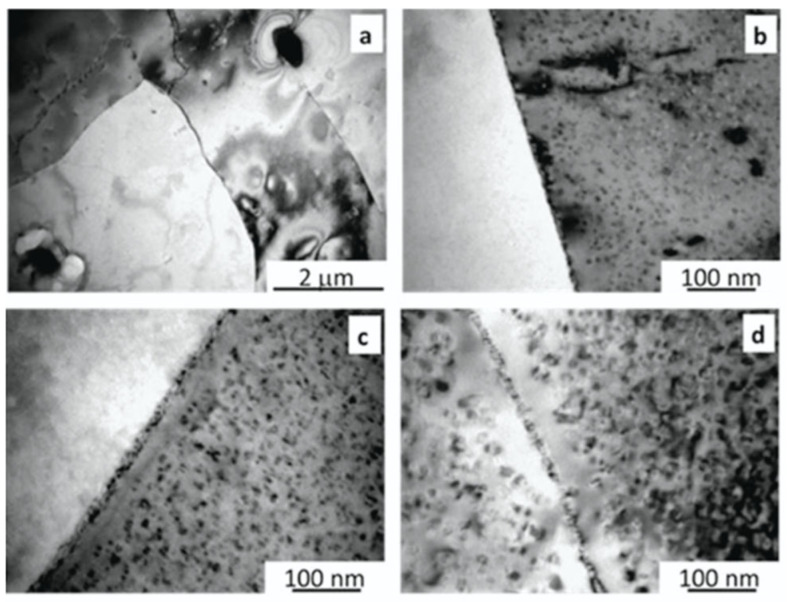
TEM pictures of (**a**) as received containing 0.4 wt% Si, (**b**) irradiated 2.73 × 10^26^ n/m^2^, E > 0.625 eV, Si content 0.68 wt% (**c**) 3.76 × 10^26^ n/m^2^, Si content 0.88 wt%, (**d**) 9.81 × 10^26^ n/m^2^, Si content 2.21 wt% [10].

TEM pictures (see Figure 3) made on HFR SURP specimens are good examples to prove this statement [11].

Cryogenic temperature irradiation slightly changes the damaged microstructure in metals. Considerable diffusion can be expected over 0.3 T_m_ (so-called homolog temperature), where T_m_ is the melting temperature in K. The melting temperature of the pure aluminum is 660 °C, and the different aluminum alloys have melting temperatures in the range of 580–660 °C; consequently, their T_m_ is in the range of −2–+25 °C). High flux fast neutron irradiation accelerates diffusion and may reduce homologous temperature. Consequently, Si precipitations caused by irradiation at low temperatures will be small and homogeneously distributed in the aluminum matrix. During the normal operational or stand-by periods of the RRs, the temperature exceeds the homologous temperature, and precipitation hardening of the AlMgSi alloy will be initiated.

### 3.4. Transmutation by Fast Neutrons

Irradiated aluminum alloys transmute under neutron flux to produce the following:

- Helium by reaction (nα) with fast neutrons;
- Hydrogen by reaction (np) with fast neutrons.

**Figure 4:**
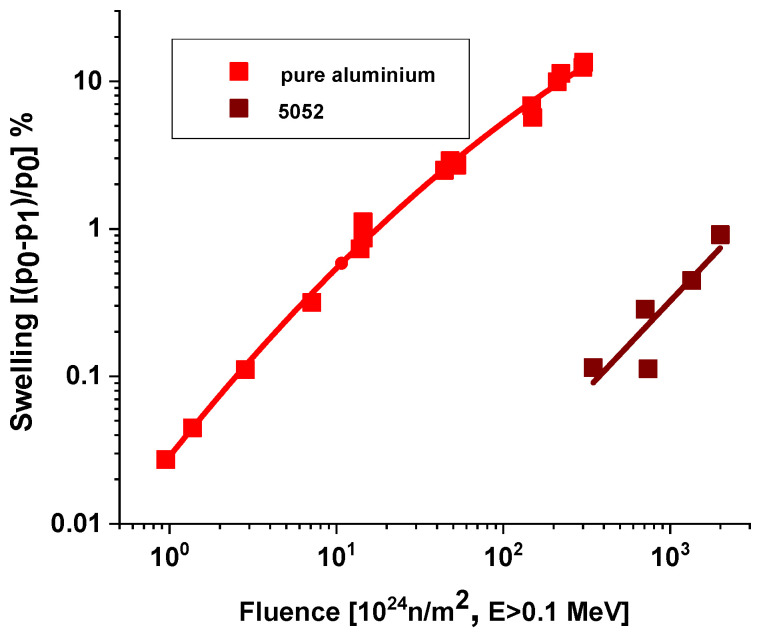
Swelling of different Al alloys [20].

Hydrogen interacts with Si, as it is a strong hydride-former. Trapping hydrogen by Si reduces gas atom mobility and further retards void nucleation. If helium and hydrogen are concentrated in voids under high pressure, they cause swelling. The silicon content of irradiated AlMg5 consumes hydrogen, and the quantity of transmuted helium is not enough to cause considerable swelling. Figure 4 shows that in the case of a 5052 alloy, the swelling threshold value is around 10^26^ n/m^2^ E > 0.1 MeV in comparison to pure aluminum, where the swelling initiated at 10^24^ n/m^2^ thermal fluence.

### 3.5. Lattice Damage Caused by Fast Neutrons

Neutron radiation effects in metals are primarily due to radiation-produced point defects, created in the lattice. Point defects are generated when an incident neutron knocks an atom from its lattice site via an elastic or inelastic collision. The recoiled atom is forced into an interstitial lattice position, known as a self-interstitial atom (SIA), and its emptied lattice site forms a vacancy. SIAs and vacancies are point defects. The kinetic energy required to recoil an aluminum atom from its site is about 25 eV. If the kinetic energy of the displaced atom is large enough, it will be transferred to many atoms and produce a cascade. One neutron with 1 MeV energy can transfer about 1.4 × 10^5^ eV to any other aluminum atom; consequently, it results in a cascade consisting of several thousand atoms.

Gamma rays are another source of atomic displacements. They do not displace atoms directly but energize electrons through Compton scattering or pair production; these electrons dislodge atoms. Such displacement events produce Frenkel pairs of SIAs and vacancies, not cascades. Gamma irradiation contributes only a few percentages compared to fast neutron radiation effects.

Loss of ductility and hardening is caused by clusters of vacancies and SIAs that obstruct glide dislocation passage during metal plastic deformation. Hardening is very sensitive to irradiation temperature because temperature is a major arbiter of cluster number and size. Lower temperatures result in more and smaller clusters; consequently, irradiation at cryogenic temperatures causes larger embrittlement than at ambient or elevated temperatures.

The operational temperature of the RRs is in the range of +20–+60 °C; thus, the operational and shut down temperature is slightly above 0.3 T_m_, and as a result a part of the matrix defects is annealed with diffusion, reducing embrittlement. Silicon atoms from transmutation, frozen into the matrix, partially combine with Mg atoms and become Mg_2_Si at room temperature, increasing embrittlement. Silicon atom diffusion without radiation is slow at room temperature.

### 3.6. Degradation of Mechanical Properties by Irradiation

The mechanical properties of AlMg and AlMgSi alloys are affected by three damage mechanisms, as discussed above:

- Matrix defects caused by fast neutrons;
- Gaseous transmutation caused by fast neutrons;
- Al-Si transmutation caused by thermal neutrons.

Irradiation effects depend on fluence, irradiation temperature, and neutron spectra type (fast/thermal neutron ratio).

Fast neutrons increase point defects. Both ductility and strength slightly increase with temperature drop, and strength decreases with increasing temperature above room temperature. However, in irradiated alloys, elevated testing temperature may cause accelerated aging (especially at high silicon and Mg_2_Si content).

**Figure 5:**
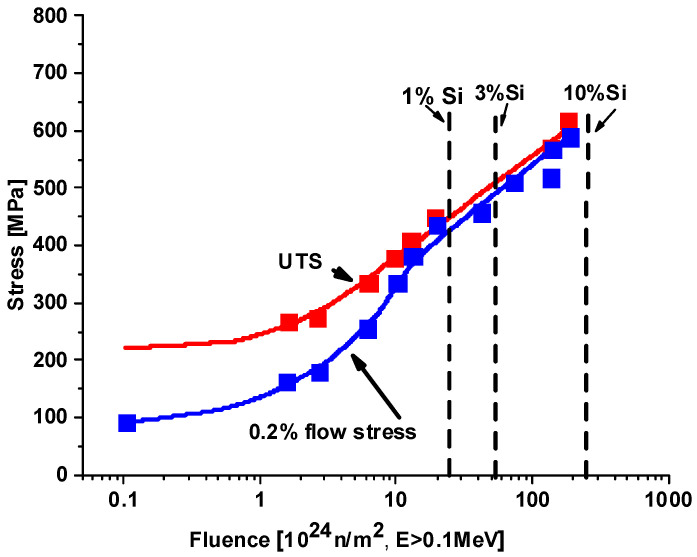
Hardening effect of high fluence irradiation on 5052 aluminum. T_irr_ = 328 K, T_test_ = 323 K, Øth/Øf = 1.7 [25,26].

Several research reactors have been built from 5xxx or 6xxx type aluminum and operated for a long time. Some parts survived fast and thermal neutron flux up to 10^26^ n/m^2^ or even beyond without failure (e.g., FRJ2, HANARO, HIFAR, BR2, LVR15, HFRs, etc. [2,20,21,22,23,24]). Figure 5 shows the effect of high fluence on the 5052 type alloy [25,26].

### 3.7. Strength Increase

Figure 5 shows test results on a high fluence irradiated 5052 alloy. Note that there is no saturation; even the 0.2 flow stress became six times larger than the non-irradiated value. The silicon content increased to nearly 10% at the highest fluence, and, still, the yield value remained slightly less than the ultimate tensile strength. This result shows that even at high silicon content and hardening, the alloy still has some ductility.

### 3.8. Reduction in Ductility

**Figure 6:**
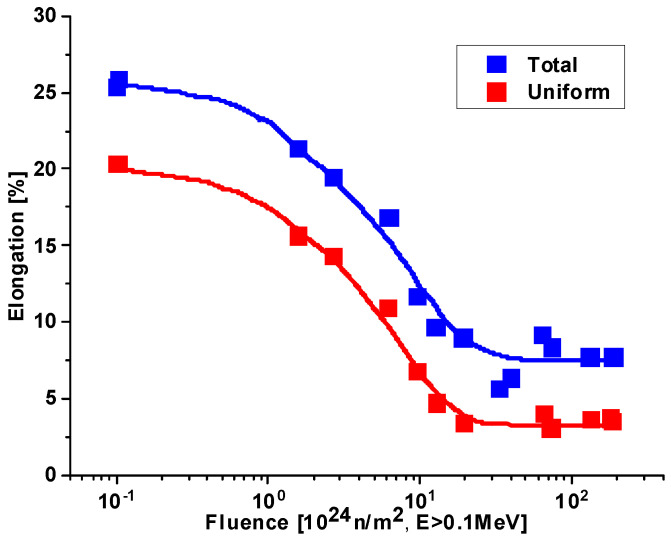
Effect of high fluence irradiation on the ductility of 5052 aluminum. T_irr_ = 328 K, T_test_ = 323 K, Øth/Øf = 1.7 [25,26].

Figure 6 introduces the elongation behavior of 5052 aluminum (the irradiated yield strength of the same material is presented in Figure 5). According to this figure, ductility is reduced, but still a considerable amount of ductility remains at the highest fluence.

OSIRIS is a French 70 MW research reactor operated at Saclay. It is used to test RPV structural materials and nuclear fuels and produces medical and industrial isotopes.

**Figure 7:**
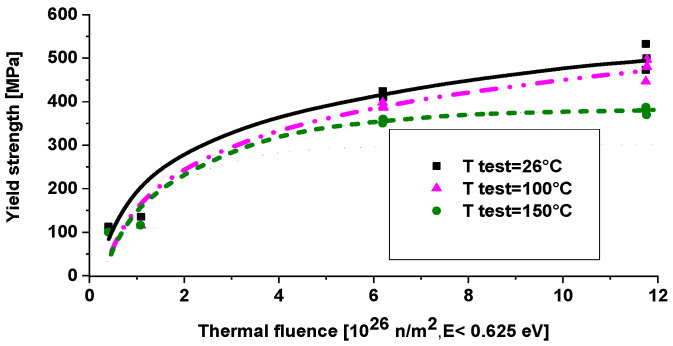
The 5754 alloy’s hardening during 30 years of operation [10].

The material properties of the OSIRIS reactor were tested after 30 years of operation. The material of it is 5754 NET alloy. The hardening during service life is given on Figure 7. Note that at room temperature testing, there is no saturation of hardening, but at higher temperatures, saturation occurs even at 6 × 10^26^ n/m^2^ thermal fluence. A similar result was obtained when testing the ORPHEE cold source shell, where the thermal/fast neutron ratio was 100. The irradiation temperature was 50 °C in both cases. Unfortunately, the testing purpose was only to select material for the Jule Horowitz reactor, so low temperature irradiated parts were not tested, or results were not published.

**Figure 8:**
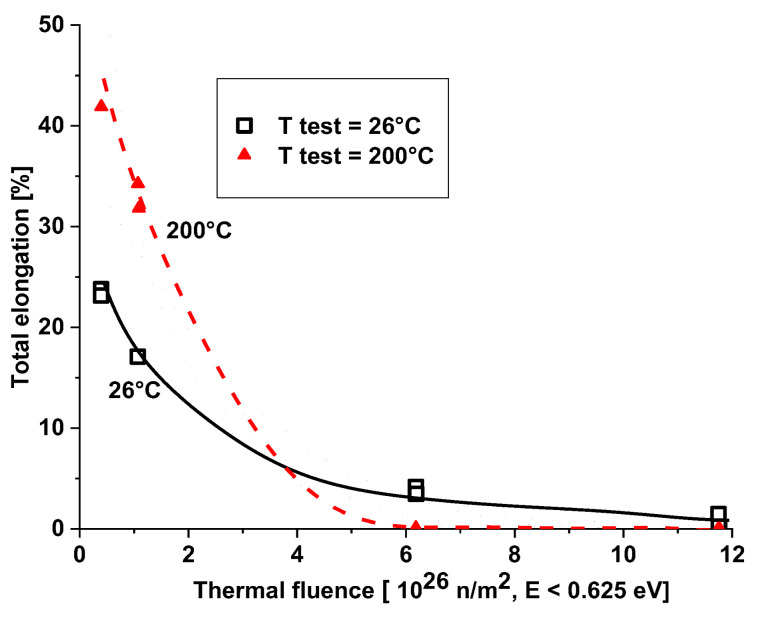
Effect of high thermal neutron irradiation on the total elongation of 5754 aluminum [9].

The 5754 alloy irradiated at about 50 °C with a conventional thermal neutron flux (E < 0.625 eV) of 2.0 × 10^18^ n/m^2^/s and a fast neutron flux (E > 1 MeV) of 2.1 × 10^18^ n/m^2^·s (0.95 thermal/fast ratio) becomes brittle at thermal fluences (E < 0.625 eV) over 11.7 × 10^26^ n/m^2^ tested at room temperature. At a thermal fluence of 6.2 × 10^26^ n/m^2^, tested at ambient temperature, the alloy shows about 4% total elongation and 3% uniform elongation, but this decreases continuously with increasing temperature. At 150 °C, the alloy becomes practically brittle at 6.2 × 10^26^ n/m^2^ fluence with a 0.2% uniform elongation and about 1.3% total elongation (see Figure 8).

The degradation depends on the activation energy of diffusion, which is the minimum energy a particle needs to move from one location to another, overcoming a potential energy barrier. It is a measure of how temperature affects the rate of diffusion; a higher activation energy means diffusion is more sensitive to temperature changes. The activation energies of volume diffusion are as follows: Al = 1.47 eV, Mg = 1.4–1.51 eV, Si = 4.24–4.76 eV [27].

Increasing the thermal/fast neutron flux ratio gives further strengthening beyond that expected from a simple increase in silicon level, only if fast flux is sufficient to activate precipitation hardening. Hence, the thermal to fast flux ratio alone is not enough to characterize aging.

**Figure 9:**
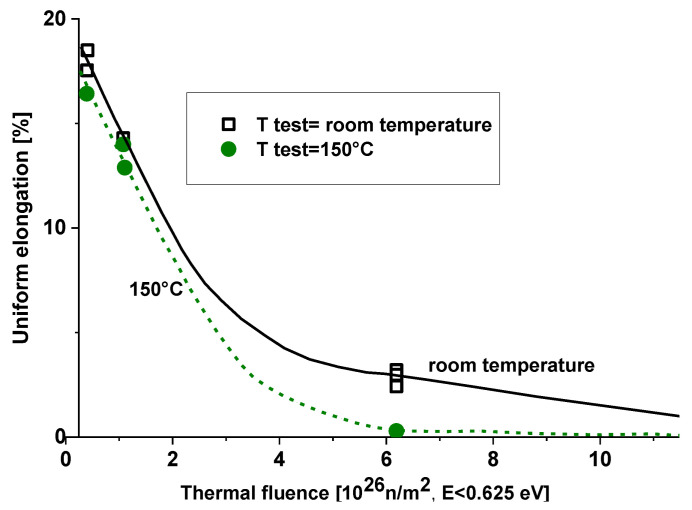
Effect of high thermal neutron irradiation on the uniform elongation of 5754 aluminum [8].

Figure 8 and Figure 9 show that some ductility remains at high thermal fluence values if the test is performed at room temperature. At elevated temperatures, the degree of precipitation hardening is expected to be high due to increased mobility, resulting in lower ductility. At room temperature, Mg_2_Si and silicon precipitations are still frozen in the matrix (diffusion is slow). During long term operation or shut down, the diffusion embrittlement partially occurs at ambient temperatures, too.

**Figure 10:**
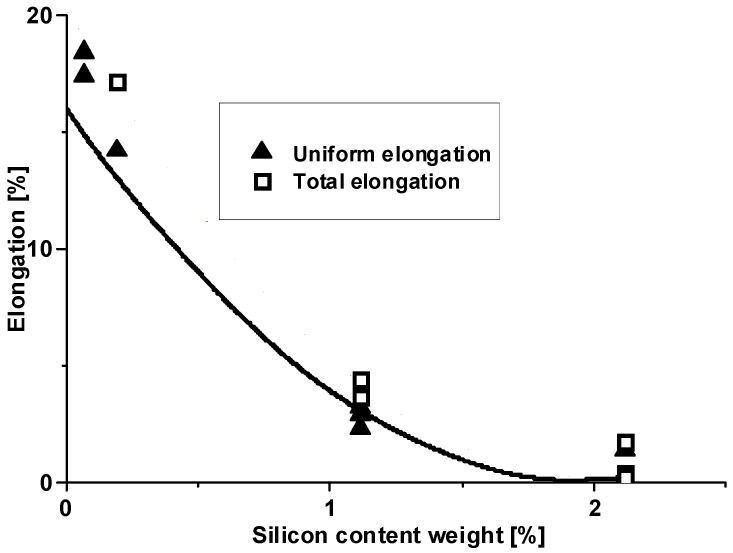
Elongation of 5754 alloy as a function of transmuted silicon content [9].

A similar result is given if elongation is plotted against silicon content for this alloy (see Figure 10). At 1.75% silicon content, ductility became zero. However, considering measured points, still 1–2% elongation can be predicted up to 2.5% silicon content. Moreover, the 5754 alloy contains about 3% Mg, so all Mg content is solved into Mg_2_Si precipitations if 1.5% silicon is transmuted. It should be noted that the ductility values measured in the 5154-O alloy used in HFR at much higher thermal fluences (and hence transmutation of Si) are higher.

### 3.9. Impact Testing

Limited data are available on the Charpy impact testing of irradiated 5xxx alloys. The Budapest Research Reactor (BRR, VVR-SZM10 type) vessel was replaced in 1988 with a new one. After operation started, a surveillance program was elaborated and carried out, where Charpy V notched specimens were cut from different places during new vessel manufacturing [28].

The base material was AlMg2.5 produced on a 99.99 clean aluminum base, but TIG welds were made with AlMg5 type filler wire. Specimens were cut from different reactor parts (such as pressure vessel wall (E), axial weldment of pressure vessel wall (V), upper zone grid (Z), and horizontal radiation channel (X)). Sixty-four specimens were tested in as received condition, and the same quantity was irradiated in one capsule at the core edge for 6216 h, resulting in a thermal fluence of 4.95 × 10^25^ n/m^2^ E < 0.625 eV). Specimens were tested at temperatures of 20 and 80 °C. The results are summarized in Table 3. Charpy impact energy slightly increased with irradiation (strengths increased faster than ductility reduced) or remained around the original value within the test result scatter. Weld impact energy is only half of the base metal value.

### 3.10. Fracture Toughness Measurements

HFR (High Fluence Research Reactor located at Petten, The Netherlands) is a tank in pool type reactor. It started operations in 1961 and usually operated more than 250 days/per year. The yearly average power is 11,655 MWday; the operating temperature is about 40 °C [29].

The old HFR vessel (Petten) made from the 5154 alloy was replaced in 1983. The maximum thermal neutron fluence at the center of the west wall was estimated at 8.3 × 10^26^ n/m^2^ (E < 0.414 eV). The corresponding silicon content was 1.9 wt%. CT specimens were cut from the material. Measured valid fracture toughness values ranged from 17.7 to 28.9 MPa√m with thermal to fast flux ratios of 1.0 and 4.8 [29].

Within this program, the irradiation effect on the fatigue crack growth rate was also measured. The irradiation effect on fatigue crack growth was found to be negligible for applied ∆K ranges between 8 MPa√m and 15 MPa√m. The measured value for ∆K = 8 MPa√m cycles was 5 × 10^−5^ mm/cycle.

**Figure 11:**
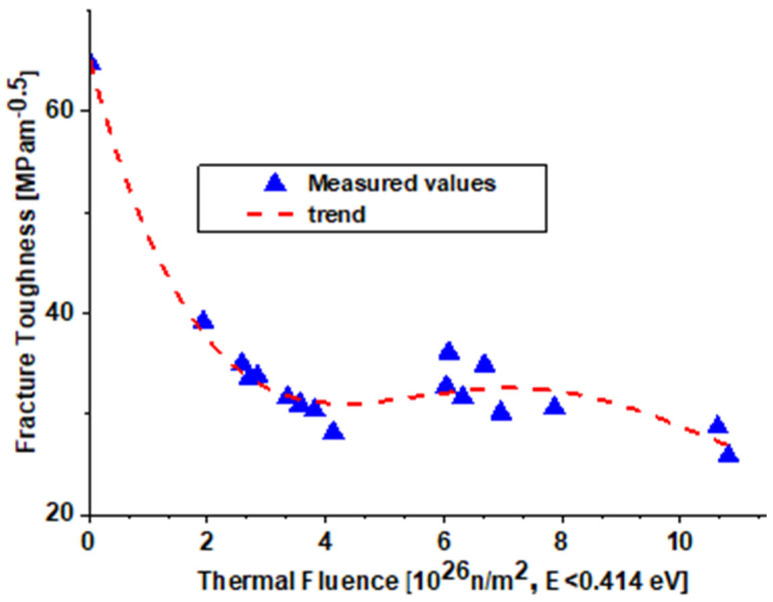
Fracture toughness values measured on the HFR material [10,11].

The reconstructed HFR is also built from the AlMg3.5 (5154-0) alloy. The reactor has a detailed surveillance program. One-inch CT specimens were irradiated and tested at room temperature, and valid J_1c_ or K_1c_ values were obtained [9,17]. Fracture toughness test results as a function of calculated thermal neutron fluence (E < 0.414 MeV) values for each tested surveillance specimen are presented in Figure 11. The fracture toughness of the unirradiated 5154-O aluminum alloy is calculated from valid J_1c_ data in accordance with the ESIS P2-92 procedure (J-R curve multiple specimen test approach). The irradiated fracture toughness data (K_IC_) presented in Figure 11 are measured and give valid results in accordance with ASTM E399-08 [30].

It should be noted that the fracture toughness value for the irradiated 5154-O aluminum alloy decreased by a factor of two compared to unirradiated material after reaching a thermal fluence value of 3 × 10^26^ n/m^2^. After this initial decrease, fracture toughness values seem to saturate at about 30 MPa√m until 10^27^ n/m^2^ thermal fluence.

## 4. Conclusions

The primary degradation mechanism affecting aluminum alloys in research reactors is radiation embrittlement, predominantly caused by transmutation due to thermal neutrons. Additionally, operational and stand-by temperatures, along with fast fluence, contribute to the degradation by increasing the diffusion rate, which subsequently alters the size and distribution of Mg_2_Si and Si precipitates.

Currently, only a limited number of research reactors have dedicated surveillance programs. For those that do not, archive materials from decommissioned units have been utilized for material testing. However, the testing methodologies employed vary significantly across different units. The MAGIC RR project seeks to address these inconsistencies by exploring the applicability of sub-size specimens for surveillance testing. The project aims to develop a best practice guide for aging management in research reactors, considering various degradation mechanisms to ensure the continued safe operation of these facilities.

This paper offers valuable insights into the aging processes affecting aluminum alloys in research reactors, which is crucial for guiding future research efforts and enhancing staff education. The findings underscore the importance of standardized testing methodologies and comprehensive surveillance programs to effectively manage the aging of materials in research reactors.

## Figures and Tables

**Table 1:** Chemical composition of the different AlMg and AlMgSi alloys.

ASTM	Mg%	Si%	Fe%	Mn%	Cu%	Zn%	Cr%
5154	3.7	0.14	0.38	0.32	0.02	0.03	0.2
5052	2–2.8	max 0.25	max 0.4	max 0.1	max 0.1	max 0.1	0.15–0.35
5083	4–4.9	max 0.4	max 0.4	0.4–1	max 0.1	max 0.25	0.05–0.25
5086	3.5–4.5	max 0.4	max 0.5	0.2–0.7	max 0.1	max 0.25	0.05–0.25
5456	4.5–5.6	0–0.3	0–0.4	0.05–0.2	max 0.1	0–0.1	0.05–0.2
5019	4.5–5.3	0–0.4	max 0.15	0.1–0.6	max 0.1	0–0.2	0–0.2
6061	0.8–1.2	0.4–0.8	0.7	0.15	max 0.1	max 0.15	0.04–0.35
6063	0.45–0.9	0.2–0.6	0.35	0.1	0.7–1.2	max 0.25	0.1

**Table 2:** Standard mechanical properties of the aluminum alloys used in RRs.

ASTM	Temper	R_p02_ MPa	UTS MPa	EL %	Temper	R_p02_ MPa	UTS MPa	El %
5154	0	min85	215–260	16	H4	>245	200	4
5052	0	min65	170–215	18	H34	180	235–285	10
5083	H111	145	300	23	H321	228	317	16
5086	H111	120	240–310	16	H14	240	300–360	3
5456	H111	131	283	15	H321	230	305–385	10
5019	H111	120	280	18	H16	290	360	2.3
6061	T4	110	180	14	T6	240	260	8
6063	T1	55	110	10	T6	170	205	8

**Table 3:** Impact testing results obtained on BRR surveillance specimens [28].

Material	Cut Location	Testing Temperature [°C]	Fluence (E < 0.625 eV) [10^25^ n/m^2^]	Absorbed Energy [J]
R-AlMg2.5 (5052)	wall	20	0	100.8
		20	4.95	100.0
		80	0	91.8
		80	4.95	92.0
R-AlMg2.5 (5052)	grid	20	0	107.3
		20	4.95	148.5
		80	0	104.0
		80	4.95	155.5
TIG welds (5456)	vessel vertical weld	20	0	60.3
		20	4.95	51.3
		80	0	56.3
		80	4.95	62.5

## Data Availability

No new data were created or analyzed in this study. Data sharing is not applicable to this article.
